# Entropy and Fractal Dimension Study of the TDP-43 Protein Low Complexity Domain Sequence in ALS Disease Severity and SARS-CoV-2 Gene Sequences in Virulence Variability

**DOI:** 10.3390/e23081038

**Published:** 2021-08-12

**Authors:** Sunil Dehipawala, Eric Cheung, George Tremberger, Tak Cheung

**Affiliations:** 1 Physics Department, City University of New York Queensborough Community College, Bayside, NY 11364, USA; sdehipawala@qcc.cuny.edu (S.D.); gtremberger@qcc.cuny.edu (G.T.); 2 Psychiatry Department, Montefiore Mount Vernon Hospital, Mount Vernon, NY 10550, USA; echeung@montefiore.org

**Keywords:** entropy, fractal dimension, TDP-43, low complexity domain sequence, SARS-CoV-2, HAR1, Znf521

## Abstract

The low complexity domain (LCD) sequence has been defined in terms of entropy using a 12 amino acid sliding window along a protein sequence in the study of disease-related genes. The amyotrophic lateral sclerosis (ALS)-related TDP-43 protein sequence with intra-LCD structural information based on cryo-EM data was published recently. An application of entropy and Higuchi fractal dimension calculations was described using the Znf521 and HAR1 sequences. A computational analysis of the intra-LCD sequence entropy and Higuchi fractal dimension values at the amino acid level and at the ATCG nucleotide level were conducted without the sliding window requirement. The computational results were consistent in predicting the intermediate entropy/fractal dimension value produced when two subsequences at two different entropy/fractal dimension values were combined. The computational method without the application of a sliding-window was extended to an analysis of the recently reported virulent genes—Orf6, Nsp6, and Orf7a—in SARS-CoV-2. The relationship between the virulence functionality and entropy values was found to have correlation coefficients between 0.84 and 0.99, using a 5% uncertainty on the cell viability data. The analysis found that the most virulent Orf6 gene sequence had the lowest nucleotide entropy and the highest protein fractal dimension, in line with extreme value theory. The Orf6 codon usage bias in relation to vaccine design was discussed.

## 1. Introduction

Studies pertaining to a low complexity domain (LCD) sequence are usually annotated with entropy values lower than a threshold, such as less than 2.2 bits per amino acid, using a 12 amino acid sliding window and consistent from the standpoint of extreme value theory [1,2]. The relationship between the entropy values of metal ion-binding protein sequences and low complexity regions has been studied [3]. For instance, the presence of multiple histidine residues in the zinc finger bonds within a protein is associated with the low complexity regions having relatively low local entropy values.

The Shannon entropy of a symbolic sequence reaches a maximum when the distribution of the symbols is uniform and where each symbol has the same probability. For the 20 amino acids that may constitute a protein sequence, the maximum entropy would be about 4.3 bits per amino acid. Similarly, for the ATCG nucleotides in an mRNA sequence listed on GenBank, the maximum mono-nucleotide entropy of a sequence would be 2 bits per nucleotide and 4 bits per di-nucleotide (AA, AT, AG, AC, TT, TC, TG, etc.). Note that the AUCG four-symbol sequence entropy calculation for a sequence in an RNA virus is the same as the ATCG four-symbol sequence entropy. The di-nucleotide entropy is nucleotide position-sensitive, while the mono-nucleotide entropy is invariant upon nucleotide shuffling, with no useful information on interactions between nucleotides. The inclusion of a sliding window analysis along an information sequence could remove the disadvantage of using position-insensitive symbols such as amino acids, nucleotides, etc. From the viewpoint of an entire sequence, a departure from a uniform distribution would generate a relatively lower entropy value, and that lower entropy value could be attributed to the utilization of a preferred symbol. It is therefore reasonable to investigate whether the low complexity domain could be effectively studied without using the 12 amino acid sliding window. This is the first question that is addressed in this exploratory study of the TDP-43 protein sequence. In the cryo-EM-based study of the TDP-43 protein folding and its association with the neuro-disease amyotrophic lateral sclerosis (ALS), the low complexity domain was thought to facilitate the specific protein assembly for disease severity [4,5].

Fractal dimension has been used for investigations in diverse fields. Fractal dimension has been used to study the scale complexity of a time series [6]. The relationship between Shannon entropy and the fractal dimension of fish trajectories has been published in Entropy [7]. The Higuchi fractal dimension of EEG signals has been shown to be useful in predicting PID-5 anxiousness [8]. The logarithmic summation in the entropy expression and the logarithmic division in the fractal dimension expression have played a role in the successful classification of molecular complexity in organic chemistry [9]. Any signals that are modeled as sequential with equal spatial intervals can be mapped onto an equivalent time series. For instance, we have previously explored the fractal dimension analysis of writing with spatial intervals [10]. By the same token, a low complexity domain or region can be investigated using fractal dimension analysis, and any relationship to entropy analysis would be illuminating. Since a protein sequence is driven by a nucleotide sequence, the low complexity domain based on entropy values of less than 2.2 in a 12 amino acid window could point to some attributes at the nucleotide level. This is the second question that is addressed in this exploratory study of the TDP-43 sequence.

Just as the low complexity domains are associated with protein folding functionality, the relatively lower entropy sequences in the SARS-CoV-2 virus could be associated with virulence variability as well. This is the third question in this exploratory study. A virulence study concluded that the three most toxic proteins associated with the virus were Orf6, Nsp6, and Orf7a [11].

## 2. Materials and Methods

The materials are sequences in the public domain. The studied TDP-43 sequences were downloaded from https://www.ncbi.nlm.nih.gov/gene/23435 (last accessed 28 June 2021). The studied SARS-CoV-2 Orf6, Nsp6, and Orf7a sequences were downloaded from https://www.ncbi.nlm.nih.gov/gene/43740572, https://www.ncbi.nlm.nih.gov/gene/43740578, https://www.ncbi.nlm.nih.gov/gene/1489674, respectively (last accessed 28 June 2021).

A nucleotide has a nucleobase, namely, cytosine-C, guanine-G, adenine-A, or thymine-T. The Shannon entropy values were computed using the p*log(p) expression, with p as the probability of an instance of a nucleobase symbol and summing over all symbols. For instance, the ATCG sequence would have an entropy maximum of 2 bits per symbol A, T, C, or G. The di-nucleotide would have an entropy maximum of 4 bits per di-nucleotide AT, AC, AG, TA, TC, TG, CA, CT, CG, GA, GT, GC, AA, TT, CC, or GG. The amino acid sequence would have an entropy maximum of about 4.32 bits per symbol, given the 20 canonical amino acids. The normalized entropy, between 0 and 1, is a measure of relative uncertainty with diverse applications, including risk analysis published in Entropy [12]. Consistent with the overall scheme of 61 codons for coding 20 amino acids and 3 codons for stopping, the normalized entropy of an amino acid sequence would be expected to be lower than that of the ATCG sequence. For instance, the Znf521 protein has been reported to be associated with psychiatric conditions [13]. The human Znf521 sequence carrying 30 zinc fingers (Gene ID 25925, 3933 ATCG nucleotide code for 1311 aa) has a mono-nucleotide entropy of 1.9923 bits (normalized entropy of 0.9961) and the amino acid sequence has an entropy of 4.1704 bits (normalized entropy of 0.9653).

There are 8 other homologous Znf521 sequences, but only 3 homolog Znf521 sequences have been verified in labs to express the protein homologs, namely, B. tarus (Bovine), M. musculus (mouse), and R. norvegicus (rat). The mouse Znf521 carrying 30 zinc fingers (Gene ID 225207) was studied. The mouse Znf521 sequence (3933 ATCG nucleotide code for 1311 aa) has a mono-nucleotide entropy of 1.9917 bits (slightly lower than that of the human sequence) and the amino acid sequence has an entropy of 4.1656 bits (slightly lower than that of the human sequence). Since the human Znf521 has 30 zinc fingers (https://www.uniprot.org/uniprot/Q96K83) (last accessed 28 June 2021) and the mouse Znf521 has 30 zinc fingers (uniprot/Q6KAS7) as well, the increase of entropy values in the ATCG nucleotide sequence and amino acid sequence in human could be independent of the incorporation of zinc fingers in the post-translation modification.

The bovine Znf521 (Gene ID 538792) carrying 24 zinc fingers was studied (uniprot/A7Z030). The bovine Znf521 sequence (3933 ATCG nucleotide code for 1311 aa) has a mono-nucleotide entropy of 1.9802 bits and the amino acid sequence has an entropy of 4.1751 bits. The rat Znf521 (Gene ID 307579) carrying 23 zinc fingers was studied (uniprot/A0A0G2JT53). The rat Znf521 sequence (3936 ATCG nucleotide code for 1312 aa) has a mono-nucleotide entropy of 1.9883 bits and the amino acid sequence has an entropy of 4.1655 bits. The additional zinc finger requirement in bovine when compared to that in rat could have suppressed the ATCG nucleotide entropy while increasing the amino acid sequence entropy in bovine.

Note that the normalized ATCG nucleotide (whole-sequence) entropy values are higher than the normalized amino acid (whole-sequence) entropy values in the studied Znf sequences in human, bovine, mouse, and rat regardless of the number of zinc fingers.

**Figure 1:**
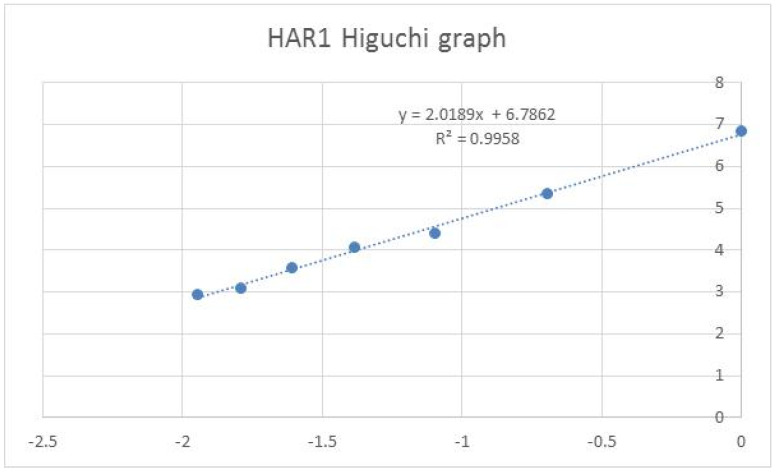
HAR1 Higuchi graph with log (L(k)) values (y-axis) versus log((1/k)) values (x-axis).

The molecular weight values of the 20 essential amino acids were used to transform the protein sequences into numeric inputs for the computation of the Higuchi fractal dimension values [6]. The proton numbers of the ATCG nucleotides, similar to molecular weight values at 5 significant figures, were used to transform the DNA sequences into numeric inputs for the computation of fractal dimension. Details of the Higuchi algorithm applications in the studies of gene sequences using a calibration procedure and of lncRNA sequences using the MATLAB procedure were reported by our group earlier [14,15]. Given a numeric series I(i) with equal intervals, a difference series (I(j) − I(i)) for different lags (j − i) could be generated. The non-normalized apparent length of the series curve is simply L(k) = Σ | I(j) − I(i)| for all (j − i) pairs that equal to k. The number of terms in a k-series would vary, and normalization must be used to obtain the series length. If the I(i) is a fractal function, then the log (L(k)) versus logα((1/k)) should be a straight line, with the slope equal to the fractal dimension. The Higuchi method incorporated a calibration division step such that the maximum theoretical value was calibrated to the value of 2. For instance, in a 1-Dim random walk model series with equal step lengths in random directions, the Higuchi fractal dimension would be 2 for the random step series; on the other hand, the random position series (Brownian motion positions with a Gaussian profile) would have a fractal dimension of 1.5, given an infinite series. The detailed calculation was provided by Higuchi [6]. All the fractal dimension values in the project were calculated using the Higuchi method and the 7-point slope, illustrated in Figure 1, was used. The programing steps were listed in our two previous publications, archived on PubMed (see References [14,15]); a computer program with a GitHub link was listed in our previous publication (see Reference [10]). The project did not study whether a sequence is a fractal object. The Excel programming steps with input/output and the GitHub link for an Excel VBA file are listed in Appendix A.

The HAR1 sequence with 118 nucleotides (nt) is the fastest evolving human sequence when compared to that of the chimpanzee. It contains 18 nucleotide substitutions or changes occurring over a span of 5 million years when comparing the human to the chimp. However, the same 118-nucleotide region only contains 2 nucleotide changes over a span of 300 million years when comparing the chimp to the chicken [16]. The increase of the CG pairs is central to one of the methylation processes in the epigenetic mechanism [17]. The change of the chimp sequence to the human HAR1 sequence could be described in terms of mutation. The Higuchi graph for HAR1 is shown in Figure 1.

**Figure 2:**
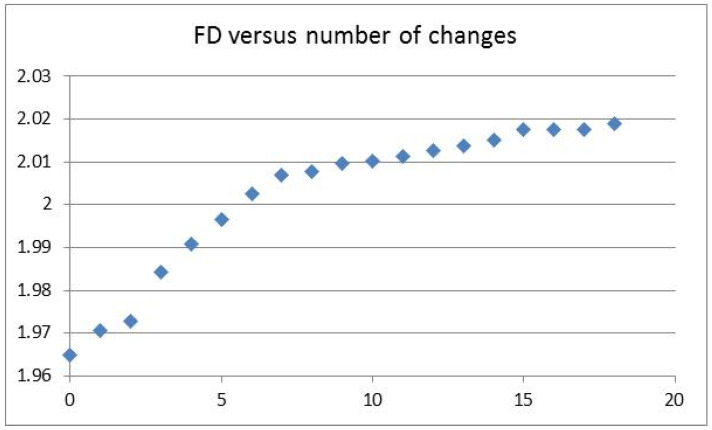
Fractal dimension FD values (y-axis) versus number of changes (x-axis).

The HAR1 sequence had a fractal dimension (FD) of 2.0189, a di-nucleotide entropy of 3.8643 bits, and a mononucleotide entropy of 1.9682 bits. The chimp counterpart sequence had an FD of 1.9649, a di-nucleotide entropy of 3.6452 bits, and a mononucleotide of 1.8625 bits. The entropy and fractal dimension values could be used to describe the 8 changes of the T-nucleotides to C-nucleotides or G-nucleotides (T >> C or G) and the 10 changes of the A-nucleotides to C-nucleotides or G-nucleotides (A >> C or G). The first chimp sequence change, using the starting position listed in Reference [16], occurred at the 6th position in which the t-nucleotide was changed to the c-nucleotide. The corresponding new entropy value Ent(1) and fractal dimension FD(1) could be computed. The last change (the chimp’s 18th change) occurred at the 113th position in which the a-nucleotide was changed to the g-nucleotide, yielding the Ent(18) and FD(18) values. The almost linear trend of the FD values could be visualized as a graph, with a different slope above FD > 2.01, as shown in Figure 2. The small dip at the second nucleotide change could be related to the description in Reference [16], which states that the 118 nt contain 2 nucleotide changes over a span of 300 million years when comparing the chimp to the chicken.

**Figure 3:**
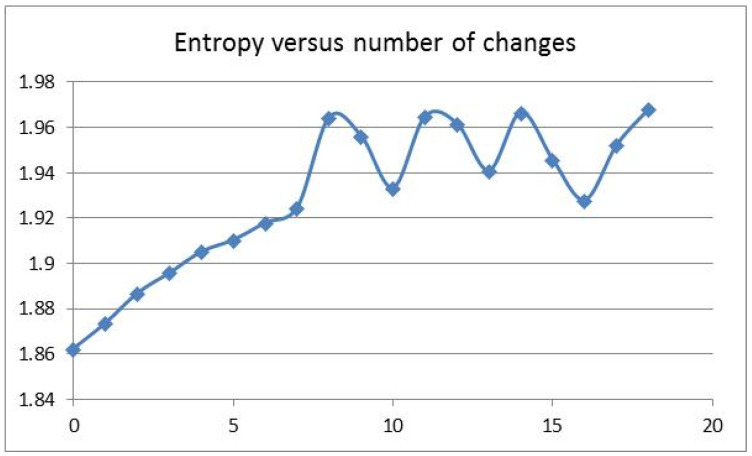
Entropy values (y-axis) versus number of changes (x-axis). The displayed line is a visual aid.

The initial linear trend of the Ent values could be visualized up to about 1.92 bits, as shown in Figure 3. The last common ancestor is an established concept in the theory of evolution. For the chimp and human, the last common ancestor could be a species having an Ent value of around 1.92 bits. The evolution toward chimp could have a gradual linear trend in Ent values, while the evolution toward human could have a fluctuating trend, starting at 1.92 bits, with an FD at 2.01, as seen in Figure 2. There was no Ent and FD correlation in the fluctuation region (Pearson correlation coefficient −0.15, N = 10). The correlation coefficient between the position-sensitive di-nucleotide entropy and the position-insensitive mono-nucleotide entropy in the fluctuation region was 0.958 (N = 11).

**Figure 4:**
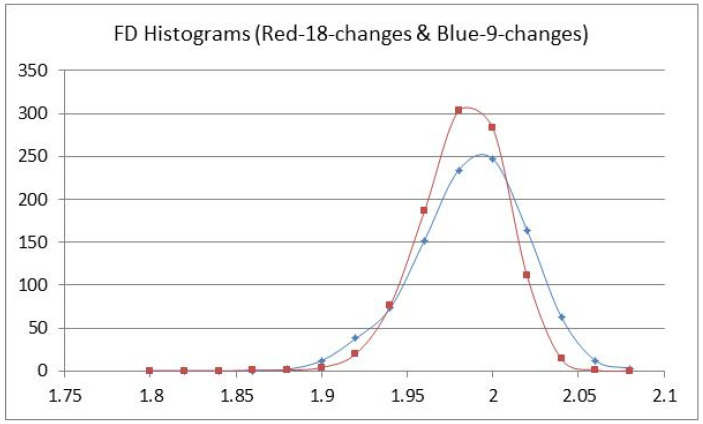
Fractal dimension histograms-18 changes (Blue) and 9 changes (Red) for N = 1000.

If the 8 T-nucleotide changes (T >> C or G) in the studied chimp sequence could occur in any of the 37 t-nucleotide positions and the 10 A-nucleotide changes (A >> C or G) in the studied chimp sequence could occur in any of the 46 A-nucleotide positions, then a simulation would reveal a histogram in which the probability of an FD > 2.02 would be about 15 percent (N = 1000). Using the histogram as a distribution, an average FD value of about 1.99 would be expected, with an uncertainty of about 0.04 using the half width, shown as the blue curve in Figure 4. If the nucleotide change had an intermediate stage of 4 T-nucleotide and 5 A-nucleotide changes, then a simulation would reveal a distribution with an FD average of about 1.98, with an uncertainty of about 0.03 using the half width, shown as the red curve in Figure 4.

**Figure 5:**
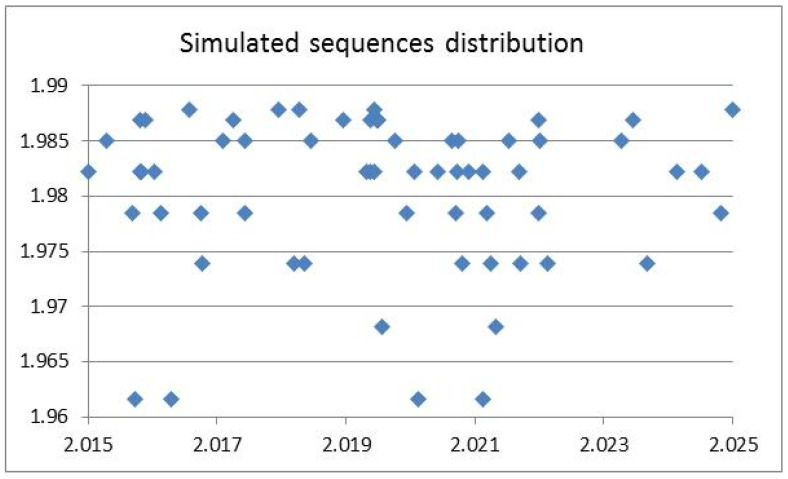
Simulated sequence distribution with entropy (y-axis) and fractal dimension (x-axis).

The histogram simulation is sensitive to the number of changes, although there was no correlation of entropy and FD in the sequences generated by the simulation. For instance, in the simulation of 1000 sequences of the 8 T-nucleotide and 10 A-nucleotide changes, there were 63 simulated sequences with FDs between 2.015 and 2.025, with a correlation of 0.004, as shown in Figure 5. Only 4 simulated sequences showed entropy values less than 1.97.

The correlation method of using entropy and FD values, and the histogram method employed in the study of the mutation phenomenon observed in HAR1 could be used to study other sequences.

Note that for the Znf521 analysis, the additional zinc finger phenomenon in bovine when compared to rat could have suppressed the ATCG nucleotide entropy while increasing the amino acid sequence FD in bovine. The rat Znf521 has an FD value of 1.9972 (ATCG entropy 1.9883 bits) and the bovine Znf521 protein has an FD value of 2.0011 (ATCG entropy of 1.9802 bits), an anti-correlation in the context of adding one zinc finger.

## 3. Results

### 3.1. Results of the TDP-43 Sequence Analysis

The entropy and fractal dimension analysis was applied to study the TDP-43 amino acid and ATCG sequences. The LCD subsequence (267–414 aa) of 3.29 bits and the non-LCD subsequence (1–266 aa) of 4.18 bits were expected to give a sequence (1–414 aa) with an in-between entropy value. The actual entropy value was 4.11 bits. This would support the use of entropy as a robust parameter for the identification of an LCD without using the 12-aa sliding window. A sliding window of 12 aa would disable any fractal dimension analysis due to the limitation of having only 12 data points. The elimination of the 12-aa sliding window would enable fractal dimension analysis while maintaining an LCD region with a low entropy value. The LCD subsequence (267–414 aa) with an FD of 2.048 and the non-LCD subsequence (1–266 aa) with an FD of 2.0243 were expected to give a sequence (1–414 aa) with an in-between FD value. The actual FD value was 2.0366. The results are listed in Table 1, showing a correlation coefficient of −0.8916 in negative correlation, inverse correlation, or anti-correlation.

Within the LCD region (267–414 aa), the cryo-EM data showed an N-terminal (276–343 aa) rich in hydrophobic amino acids with mostly short beta strands, independent of the steric-zipper interactions. This 68 amino acid subsequence (276–343 aa) within the LCD sequence had the lowest entropy of 3.09 bits. The remaining LCD subsequence of 71 amino acid (344–414 aa) had an entropy of 3.16 bits. However, the combined (276–343 aa) and (344–414 aa) sequences had an entropy of 3.22 bits, which was outside the range of 3.09–3.16 bits. The correlation coefficient of −0.111 in Table 1 showed that the protein entropy parameter was not useful for intra-LCD sequence comparison in the case of TDP-43 protein analysis.

Since proteins are generated from ATCG sequences, it would be instructional to study the corresponding ATCG sequences in the framework of intra-LCD analysis. Trends in both entropy and fractal dimension values were observed in the intra-LCD data, shown in Table 2. Thus, the answer to the second research question was affirmative. The intra-LCD amino acid entropy values were consistent with the ATCG nucleotide entropy values.

The ATCG data showed that di-nucleotide entropy correlated with fractal dimension. The first three rows in Table 2 were the results using the entire TDP-43 sequence. The entire TDP-43 sequence (1–414 aa) was coded by 1242 ATCG nucleotide (nt). The TDP-43 beginning sequence (1–266 aa) was coded by 798 nt. The TDP-43 LCD sequence (267–414 aa) was coded by 444 nt. The correlation coefficient between the FD and di-nucleotide entropy was 0.9931, and the correlation coefficient between FD and mono nucleotide entropy was 0.9970, as shown in Table 2, with positive correlations.

For the intra-LCD analysis using the cryo-EM data, the ATCG data showed that di-nucleotide entropy correlated with the fractal dimension. The N-terminal LCD sequence (276–343 aa) was coded by 204 nt, the remaining LCD sequence (344–414 aa) was coded by 213 nt, and the combined sequence (276–414 aa) was coded by 417 ATCG nt. The correlation coefficients between the FD and entropy are shown in Table 2, with positive correlations.

### 3.2. Results of the SARS-CoV-2 Virulent Sequence Analysis

A similar fractal dimension analysis was performed on the SARS-CoV-2 protein sequences, on virulence versus fractal dimension. The studied proteins were Orf6, Nsp6, and Orf7a, which are the three most toxic proteins indicated by the cell viability data. The cell viability data were obtained from Reference [11], Figure 1. The cell viability data were estimated to have an uncertainty of about 5%. There are 61 aa in Orf6, 290 aa in Nsp6, and 122 aa in Orf7a. The correlation coefficient between the amino acid sequence FD and the cell viability data varied from −0.9020 to −0.9999, as shown in Table 3, and was given an estimated uncertainty of 5% in the cell viability data.

A similar entropy calculation was performed on the SARS-CoV-2 ATCG nucleotide sequences listed on GenBank. Note that the AUCG four-symbol sequence entropy calculation for an RNA virus is the same as the entropy of the ATCG sequence listed on GenBank. The 61-aa Orf6 protein sequence was coded by a corresponding 183 nt sequence. The 290-aa Nsp6 protein sequence was coded by a corresponding 870 nt sequence. The 122-aa Orf7a protein sequence was coded by a corresponding 366 nt sequence. The correlation coefficient between the ATCG/AUCG sequence di-nucleotide entropy and the cell viability varied from 0.8544 to 0.9999, as shown in Table 3. The correlation coefficient between the ATCG/AUCG sequence mono-nucleotide entropy and the cell viability data varied from 0.8538 to 0.9993, shown in Table 3, and given an estimated uncertainty of 5% in the viability data.

Highlights of the results are summarized as follows. The Orf6, Nsp6, and Orf7a sequences in SARS-CoV-2 were analyzed, guided by a recent report on the level of virulence associated with the three most toxic proteins, as indicated by the cell viability data [11]. The functional informatics in the studied SARS-CoV-2 sequences were shown to be associated with virulence functionality, with absolute correlation coefficient values from 0.84 to 0.99, given an uncertainty of 5% in the cell viability data. The results suggest an affirmative answer to the third question in this study, that the SARS-CoV-2 virulence variability is associated with low Shannon entropy values of the ATCG/AUCG sequences.

The ATCG/AUCG sequences carry the information for the amino acid products. The reduction of uncertainty in the amino acid sequences with lower normalized Shannon entropy values are shown to have ratio values less than one, displayed in Table 4. The studied sequences’ correlation coefficients of ATCG/AUCG entropy and amino acid FD are shown in Table 5, grouped according to virulence functionality. Note that the fractal dimension values of the SARS protein sequences were used in the analysis and shown in Table 5, but not the fractal dimension of the SARS AUCG sequences. The fractal dimension analysis on the studied AUCG sequences was not included in the project, partly due to a lack of PubMed references (as far as we know).

## 4. Discussion

The results presented here support the view that Shannon entropy is a robust parameter in the study of the TDP-43 ATCG nucleotide and amino acid protein sequences. Its application to the study of SARS-CoV-2 virulence in terms of the three most toxic sequences showed correlation. The findings from studies using micro-RNA to predict which human genes are susceptible to infection could be used to examine the entropy–fractal dimension relationship that pertains to those genes [18,19]. Limitations to the present study include, for example, the absence of a correlation study of the entropy values and protein folding structures, and the absence of a correlation study of entropy values and nucleotide binding energy. The study of di-amino-acid entropy, with 210 outcomes for the typical 20 amino acids, could be applied to long protein sequences. The use of fractal dimension to supplement the entropy analysis of intra-LCD sequences in other genes would be instructive as well. The significance of extreme value theory in the context of virulence functionality could be elucidated further in terms of entropy and fractal dimension values in future studies of various viruses.

The present study showed that the lowest ATCG/AUCG di-nucleotide entropy value and the highest amino acid protein fractal dimension value of the SARS-CoV-2 Orf6 gene are associated with marked virulence. A conjecture maintaining that extreme values must be related to extreme virulence could be formulated as follows: A relatively low entropy nucleotide sequence with a relatively high fractal dimension protein sequence could be a very specific event in the evolution of the Orf6 gene. Such a conjecture may be applicable to the study of Orf6 evolution with applications [20,21].

The bias associated with human codon usage where the third base (GC3) favors stabilization and (AT3) favors destabilization could be relevant to the study of the virulence functionality of Orf6 and other genes [22]. The Orf6 186 nt sequence has 19 (1st, 2nd, A) codons, 25 (1st, 2nd, C) codons, 7 (1st, 2nd, G) codons, and 11 (1st, 2nd, C) codons, totaling 61 codons and one stop codon that enable a ribosome to generate 61 aa residues. A high tendency toward destabilization, which would imply low optimality with the greatest virulence functionality in Orf6, could be interpreted as an apparent paradox [23]. The vaccine design principle based on protein synthesis, with modulation controlled by codon usage bias [24], could be used against the Orf6 and other sequences associated with virulence. The extreme entropy and fractal dimension values observed in the Orf6 sequence could be elucidated in future nucleotide substitution studies that assess cell viability and codon usage bias. The results of such studies could also provide selection criteria for designing vaccines with optimum efficacy and precision delivery.

Scientists have studied entropy for many years. The entropy inside a living cell can be reduced when the cell’s DNA instructions call for it. This decrease in entropy and its associated increase in the cell’s environment are consistent with the physical laws of entropy. The extreme value theory perspective could be used to study an initial condition, which would differ from the Goldilocks principle where balancing over time is the central mechanism. The extremely low entropy value of the Orf6 sequence could be indicative of a special initial condition. Among the studies on the Big Bang’s initial conditions, Penrose found that an extreme accuracy of one part in 10 to the power of 10^123, from an entropy of 10^123 in natural units, would be required in order to make the present universe compatible with the second law of thermodynamics [25,26]. Perlmutter cited the extreme value of the cosmological constant, 10^−122 Planck’s length squared, as evidence of the existence of other models beyond the standard model of particle physics in the discussion of fine tuning [27,28]. The physics approach in the study of change includes the use of a differential equation process in which an input of initial and/or boundary conditions is required. Similarly, the biology approach in the study of change includes the process of evolution in which DNA/RNA informatics is required. The extreme value theory perspective, in view of the extreme entropy and fractal dimension values of the Orf6 sequence, could enter into the discussion of initial fine tuning in terms of DNA informatics with their initial selections, if any, in future studies.

Alan Guth stated that the one-time event of Big Bang would not preclude probabilistic modeling [29]. Guth emphasized that Cosmology studies on the one-time Big Bang event shows that there are quantities that we can probe with high certainty, but there are cosmic variants with high uncertainty. It would be instructive to illustrate a probabilistic perspective via the extreme value theory if the extreme low entropy value of the Orf6 sequence could be indicative of a special initial condition—perhaps very special, in our opinion.

## 5. Conclusions

In conclusion, this exploratory research supported the extreme value theory in terms of entropy for sequence analysis and in relation to functionality; this was built on the examples of the TDP-43 sequence with its low complexity domain and the extreme virulence of the SARS-CoV-2 sequence. The identification of a low complexity domain using the 12 amino acid sliding window could be supplemented with a fractal dimension analysis of the entire domain, if the entire domain also exhibits low Shannon entropy without using the 12 amino acid window. Adding fractal dimension analysis to low complexity domain analysis would necessitate the removal of the requirement of a 12 amino acid sliding window.

Since a protein sequence is coded by a nucleotide sequence, the low complexity domain based on entropy values of less than 2.2 in a 12 amino acid window would point to some structures at the nucleotide level. This reasonable working hypothesis allows the ATCG sequence of a low complexity domain to be amenable to evolutionary research with nucleotide substitutions, similar to the HAR1 example detailed in the Methods section.

Just as the low complexity domains are associated with protein folding functionality in cryo-EM data, the relatively low entropy sequences in the SARS-CoV-2 virus could be associated with virulence variability as well. The analysis in this report supported such an association, namely, the three most toxic proteins Orf6, Nsp6, and Orf7a demonstrated a correlation between virulence variability and sequence entropy, with correlation coefficient values from 0.84 to 0.99 due to an estimation of 5% uncertainty in the available cell viability data from a recent publication [11].

The HAR1 analysis offered an instructional methodology. The linear trend for entropy (bits per nucleotide change) and the subsequent fluctuation could be indicative of the existence of the last common ancestor of chimp and human. The fluctuation feature in entropy could be described by a smaller FD slope (FD per nucleotide change) when FD > 2.01. The nucleotide change in the environment of stable neighboring nucleotides was modeled as the basic driving mechanism, which could be represented by the two linear slopes for chimp and human, respectively. On the one hand, the entropy fluctuation slope after 1.92 bits (with smaller FD) in the HAR1 analysis could be indicative of a different selection pressure for human when compared to the chimp. On the other hand, if the last common ancestor had an Ent value less than 1.92 bits, for instance, an Ent value of 1.89 bits in the second nucleotide change followed by the signature of a small FD value dip at around 1.972, then a transition from the linear trend to fluctuation would be seen in the entropy values in the evolution toward human. In such a transition scenario, the second slope in the fractal dimension could serve as an ordering parameter for the entropy fluctuation beyond the transition. Furthermore, the rate of the change of the slope (FD per nucleotide change) could be an indicator of an associated phase transition, similar to the change of magnetic susceptibility (magnetization per applied field change) in the magnetic phase transition theory. Future data on the last common ancestor of chimp and human would elucidate the HAR1 instructional methodology.

The anti-correlation with a negative correlation coefficient in the protein FD and the ATCG/AUCG entropy parameters for the SARS-CoV-2 sequences are interesting phenomena. The functional information embedded in RNA is used to generate amino acids with folding interaction in order to respond to an environment. The reduction of uncertainty in ATCG/AUCG sequences indicated by the Shannon entropy could be necessary to generate the complexity indicated by the FD of the product proteins, grouped by specific functionality, namely, the virulence variability in the case of SARS-CoV-2. In the context of adding one zinc finger to the bovine Znf521 (24 zinc fingers), when compared to the rat Znf521 (23 zinc fingers), a decrease in ATCG entropy along with an increase in the product protein FD was also found.

The SARS-CoV-2 CGG-CGG codon pairs needed for the construction of the RR (Arginine doublet) in the PRRAR motif for the furin-cleavage site, which enhances the docking to the human cell membrane, have been accepted as an extremely rare codon usage (a unique recombination occurrence) in CoVs [30]. The recent pending-review conclusion of “difficult to consider a virus recombination as mechanism for the PRRA acquisition” would deserve further analysis [31]. The present study would provide a justification for the future study of the evolution of the SARS-CoV-2 using the method of entropy and fractal dimension, as illustrated by the analyses on Znf521, HAR1, and the cryo-EM identified subsequences in the LCD inside the TDP-43 protein.

On the one hand, the existence of functional proteins with the information property of higher normalized Shannon entropy in the amino acid sequences as compared to the nucleotide sequences could be an interesting investigation in terms of recombination events, laboratory constructions, etc. On the other hand, a higher normalized Shannon entropy value in the nucleotide sequence could be indicative of the nature of a dynamical functional module, similar to that proposed in the study comparing E-coli coding to the Linux operating system of a computer [32]. The methylations of A-nucleotide and CG pair at specific sites could be counted as new varieties in a nucleotide sequence such that the normalized Shannon entropy could change. The epigenetic data showing that the studied centenarians have a lower DNA methylation content when compared to studied newborns could support a hypothesis that the aging process could be marked by changes in the normalized Shannon entropy [33].

For the studied TDP-43 DNA sequences and the resulting amino acid sequences, there are positive correlations between fractal dimension and entropy values. Table 1 shows a positive correlation coefficient in the studied TDP-43 protein sequences, grouped according to the functionality of LCD but not the functionality of the N-terminal using cryo-EM. Table 2 shows positive correlation coefficients in the studied TDP-43 ATCG sequence, grouped according to the functionality of generating the corresponding mRNA sequences.

The project did not find a set of correlation coefficient values between entropy and fractal dimension in all of the studied ATCG coding sequences and amino acid product sequences. There were low correlation coefficient values. For instance, the TDP-43 sequences within the LCD sub-regions discovered by cryo-EM showed a low correlation coefficient, as shown in Table 3. A set of studied sequences showing correlation are within a special functionality such as the coding sequence (DNA CDS) to support LCD functionality in TDP-43, the virulence functionality in SARS-CoV-2, etc. A “special” functionality revealed by a correlation would suggest an interrelation between the probabilities and fractal dimension, and point to an underlying mechanism waiting to be discovered. Laboratory tools such as recombination, substitution, etc., could be used experimentally to elucidate the underlying mechanism in the theory of evolution. The project results suggest that those “special” sequences with a high correlation would offer better opportunities in laboratory investigations when compared to other sequences showing a low correlation, grouped according to functionality. For instance, with the use of programming tools, an interpolation estimation of the fractal dimension values could generate a “gain in function” or “loss in function”, given an observed association with a specific functionality. Note that the correlation coefficient would not reflect nonlinear relationships; as a counterexample, a strong cyclic (or seasonal) nonlinear association could have a small correlation coefficient [34]. Within a linear-association working hypothesis, knowing the association of the fractal dimension values with the entropy values built from the underlying probability profiles could serve as a guide to the use of the laboratory tools. The extrapolation estimation scheme could have far-reaching consequences in terms of the “gain in function”, understanding of the mutations in evolutionary theory, etc., if an interpolation investigation within a linear-association working hypothesis is successful.

## Figures and Tables

**Table 1:** Results of the studied TDP-43 amino acid sequences.

TDP-43 Protein Sequences	Fractal Dimension (Amino Acid mol wt)	Amino Acid Entropy	Correlation Coefficient	Annotations
266 aa (1–266)	2.0243	4.1769	−0.8916 anti-corr	Non-LCD
148 aa (267–414)	2.0483	3.2918		LCD inside TDP-43
414 aa	2.0366	4.1098		entire TDP-43
68 aa (276–343)	2.0588	3.0859	−0.111 no correlation	cryo-EM N-terminal of LCD
71 aa (344–414)	2.0443	3.1614		cryo-EM Remaining LCD
139 aa (276–414)	2.0580	3.2221		cryo-EM LCD

**Table 2:** Results of the studied TDP-43 ATCG sequences.

TDP-43 Protein Sequences	Fractal Dimension ATCG	ATCG Di-Nucleotide Entropy (4 Bits Max)	ATCG Mono-Nucleotide Entropy (2 Bits Max)	Correlation Coefficient	Annotations
266 aa (1–266)	1.9939	3.9278	1.9812	0.9931 (FD and di-nucleotide entropy)	Non-LCD
148 aa (267–414)	1.9757	3.8367	1.9665	0.9970 (FD and mono-nucleotide entropy)	LCD inside TDP-43
414 aa	1.9892	3.9146	1.9785		entire TDP-43
68 aa (276–343)	1.9652	3.7538	1.9378	0.9233 (FD and di-nucleotide entropy)	cryo-EM N-terminal of LCD
71 aa (344–414)	1.9833	3.8528	1.9896	0.9731 (FD and mono-nucleotide entropy)	cryo-EM Remaining LCD
139 aa (276–414)	1.9731	3.8318	1.9709		cryo-EM LCD

**Table 3:** Results of the studied SARS-CoV-2 sequences.

SARS-CoV-2 Sequences	FD Amino Acid (Using mol wt)	Cell Viability Min (arb. Units)	Cell Viability Max (arb. Units)	Correlation Coeficient (Min to Max)
Orf6 (61 aa)	2.0242	110.2	121.8	−0.99 to −0.90 anti-corr
Nsp6 (290 aa)	1.9743	138.7	153.3	
Orf7a (122 aa)	1.9594	151.05	166.95	
	Amino acid entropy (using mol wt)			
Orf6 (61 aa)	3.8553	110.2	121.8	0.7026 to 0.998
Nsp6 (290 aa)	4.0707	138.7	153.3	
Orf7a (122 aa)	4.0418	151.05	166.95	
	di-nucleotide ATCG/AUCG entropy			
Orf6 (183 nt)	3.6756	110.2	121.8	0.8444 to 0.9999
Nsp6 (870 nt)	3.7715	138.7	153.3	
Orf7a (366 nt)	3.8660	151.05	166.95	
	mono-nucleotide ATCG/AUCG entropy			
Orf6 (183 nt)	1.8610	110.2	121.8	0.8538 to 0.9992
Nsp6 (870 nt)	1.9161	138.7	153.3	
Orf7a (366 nt)	1.9640	151.05	166.95	

**Table 4:** Normalized Shannon entropy of the studied SARS-CoV-2 sequences.

SARS-CoV-2 Sequences	Normalized Shannon Entropy Amino Acid Seq/4.32 Bits	Normalized Shannon Di-Nucleotide ATCG(AUCG) Entropy/4 Bits	Ratio of Normalized Shannon Entropy-Amino Acid Seq/Nucleotide Seq
Orf6 (61 aa)	3.8553/4.32	3.6756/4	0.9712
Nsp6 (290 aa)	4.0707/4.32	3.7715/4	0.9994
Orf7a (122 aa)	4.0418/4.32	3.8660/4	0.9680
		Normalized Shannon mono-nucleotide ATCG(AUCG) entropy/2 bits	
Orf6 (183 nt)	3.8553/4.32	1.8610/2	0.9591
Nsp6 (870 nt)	4.0707/4.32	1.9161/2	0.9836
Orf7a (366 nt)	4.0418/4.32	1.9640/2	0.9528

**Table 5:** Correlations of the studied SARS-CoV-2 sequences.

SARS-CoV-2 Sequences	FD Amino Acid Seq (Using mol wt)	Di-Nucleotide ATCG(AUCG) Entropy	Correlation Coefficient
Orf6 (61 aa)	2.0242	3.6756	−0.9559 anti-corr
Nsp6 (290 aa)	1.9743	3.7715	
Orf7a (122 aa)	1.9594	3.8660	
		mono-nucleotide ATCG(AUCG) entropy	
Orf6 (183 nt)	2.0242	1.8610	−0.9659 anti-corr
Nsp6 (870 nt)	1.9743	1.9161	
Orf7a (366 nt)	1.9594	1.9640	

## Data Availability

All of the data used were in the public domain.

## Appendix A

Excel VBA command lines for Higuchi fractal dimension calculation

‘data in price(i) array

For i = 1 To NN   ’calculation starts

DST(i) = price(i)

sumdst = sumdst + DST(i)

IMF(i) = price(i)

sumimf = sumimf + IMF(i)

Next

avgdst = sumdst/NN

avgimf = sumimf/NN

For i = 1 To NN

vardst = vardst + (DST(i) − avgdst)^2

varimf = varimf + (IMF(i) − avgimf)^2

Next

For k = 1 To NN

For M = 1 To k

For i = 1 To Int((NN − M)/k)

sp = sp + (Abs((DST(k * i + M) − IMF(k * (i − 1) + M)))) * (NN − 1)/(k * (Int((NN − M)/k)))

Next i

r(k) = r(k) + sp

sp = 0

Next M

r(k) = r(k)/k/k

r(k) = (Log(r(k) + 0.00000001)) ’ log to base e

Next k

‘calc fractal 7-point slope

ss = 7

For i = 1 To ss

avgr = avgr + r(i)

avglog = avglog + Log(1/i)

Next

avgr = avgr/ss

avglog = avglog/ss

For i = 1 To ss

slope1 = slope1 + (r(i) − avgr) * (Log(1/i) − avglog)

slope2 = slope2 + (Log(1/i) − avglog)^2

Next

Slope = slope1/slope2

‘Print “7-pt-slope=”; Slope

CommandButton3.Caption = “7-pt-slope=” + Str$(Slope)

An Excel VBA program for the Higuchi fractal dimension calculation has been uploaded onto GitHub, https://github.com/taklingustics/taklingustics, (last accessed 2 August 2021) for interested readers.
